# Body temperature and cold sensation during and following exercise under temperate room conditions in cold‐sensitive young trained females

**DOI:** 10.14814/phy2.13465

**Published:** 2017-10-23

**Authors:** Naoto Fujii, Erii Aoki‐Murakami, Bun Tsuji, Glen P. Kenny, Kei Nagashima, Narihiko Kondo, Takeshi Nishiyasu

**Affiliations:** ^1^ Faculty of Health and Sport Sciences University of Tsukuba Tsukuba City Japan; ^2^ Human and Environmental Physiology Research Unit University of Ottawa Ottawa Canada; ^3^ Faculty of Human Culture and Science Prefectural University of Hiroshima Hiroshima Japan; ^4^ Body Temperature and Fluid Laboratory (Laboratory of Integrative Physiology) Faculty of Human Sciences Waseda University Tokorozawa Japan; ^5^ Graduate School of Human Development and Environment Kobe University Kobe Japan; ^6^ Present address: The University of Tsukuba Faculty of Health and Sport Sciences Tsukuba City Japan

**Keywords:** Behavioral thermoregulation, Cold disorder, perceptual thermal sensitivity, thermal comfort

## Abstract

We evaluated cold sensation at rest and in response to exercise‐induced changes in core and skin temperatures in cold‐sensitive exercise trained females. Fifty‐eight trained young females were screened by a questionnaire, selecting cold‐sensitive (Cold‐sensitive, *n* = 7) and non‐cold‐sensitive (Control, *n* = 7) individuals. Participants rested in a room at 29.5°C for ~100 min after which ambient temperature was reduced to 23.5°C where they remained resting for 60 min. Participants then performed 30‐min of moderate intensity cycling (50% peak oxygen uptake) followed by a 60‐min recovery. Core and mean skin temperatures and cold sensation over the whole‐body and extremities (fingers and toes) were assessed throughout. Resting core temperature was lower in the Cold‐sensitive relative to Control group (36.4 ± 0.3 vs. 36.7 ± 0.2°C). Core temperature increased to similar levels at end‐exercise (~37.2°C) and gradually returned to near preexercise rest levels at the end of recovery (>36.6°C). Whole‐body cold sensation was greater in the Cold‐sensitive relative to Control group during resting at a room temperature of 23.5°C only without a difference in mean skin temperature between groups. In contrast, cold sensation of the extremities was greater in the Cold‐sensitive group prior to, during and following exercise albeit this was not paralleled by differences in mean extremity skin temperature. We show that young trained females who are sensitive to cold exhibit augmented whole‐body cold sensation during rest under temperate ambient conditions. However, this response is diminished during and following exercise. In contrast, cold sensation of extremities is augmented during resting that persists during and following exercise.

## Introduction

Cold exposure is associated with an enhanced cold sensation that is thought to be primarily mediated by reductions in skin temperatures (Gagge et al. 1969; Attia 1984; Katsuura et al. 1998). The magnitude of increase in cold sensation in response to reductions in skin temperatures differs between males and females such that females demonstrate a greater cold sensation relative to their male counterparts (Krauchi et al. 2008; Mozaffarieh et al. 2010; Pham et al. 2016).

When exposed to a thermoneutral ambient temperature condition while fully clothed (i.e., ~25°C) most individuals do not feel cold. However, some individuals, typically young females, demonstrate a strong cold sensation; a response which has been termed “hi‐e‐sho” in Japanese (American Society of Heating 1972). Nagashima et al. (2002) demonstrated that young females who are sensitive to cold exhibit greater cold sensation over the whole body as well as the extremities (i.e., fingers and toes) during resting in a room temperature of 23.5°C while donning a sleeveless shirt and short pants. The augmented cold sensation was found to be associated with greater cold sensitivity in response to decreasing skin temperatures (Nagashima et al. 2002), a response which was subsequently confirmed by others (Yamazaki 2015; Yamazaki and Sone 2017). It is important to note that these previous studies examined physically inactive young females (Nagashima et al. 2002; Yamazaki 2015; Yamazaki and Sone 2017). As such, it remains to be determined whether similar responses are observed in young females who are regularly engaged in physical training.

For maintaining health or improving athletic performance in competitive sporting events, many individuals exercise in the winter season outdoors. In addition to participating in traditional winter sports (i.e., skiing, skating, others), individuals may participate in non‐winter sporting events such as running marathons which are often held in the winter season. Exercise has been shown to blunt thermal sensation to both cold and heat exposure (i.e., less sensitive to cold and heat) (Kemppainen et al. 1985; Ouzzahra et al. 2012; Gerrett et al. 2014, 2015; Flouris and Schlader 2015). Also, performing exercise induces a greater rate of metabolic heat production in the active muscles that warms the body causing rapid increases in body temperatures (i.e., muscle, core, and skin) (Kenny and Jay 2013). In the study by Nagashima et al. (2002), while cold‐sensitive young females exhibit greater cold sensation during exposure to a non‐heat stress room temperature of 23.5°C under resting conditions, this response becomes diminished as skin temperature increases. It is plausible therefore that the increase in skin temperature that accompanies an exercise‐induced heat stress may diminish augmented cold sensation in young trained females who have a high sensitivity to cold. To the best of our knowledge no study has evaluated how thermal sensation may be altered, if at all, by a prior bout of exercise. It is well documented that body heat storage remains significantly elevated for a prolonged period (60–90 min) following cessation of exercise as evidenced by sustained elevations in muscle, core and skin temperatures that endures for an extended period (60–90 min) (Kenny and McGinn 2017). It is possible therefore that an exercise‐mediated change in cold sensation in young trained females may remain intact for a prolonged period following cessation of exercise.

The purpose of this study was therefore to examine cold sensation at rest as well as during and following an exercise‐induced increase in body temperature in cold‐sensitive young exercise trained females under temperate ambient conditions (i.e., 23.5°C). We hypothesized that young exercise trained females who are sensitive to cold will exhibit greater cold sensation during exposure to temperate ambient conditions at rest as compared to their non‐cold‐sensitive counterparts. However, this response would be diminished during and following an exercise‐induced heat stress.

## Materials and Methods

### Ethical approval

This study was approved by the Human Subjects Committee of the University of Tsukuba, in agreement with the Declaration of Helsinki. Written informed consent was obtained from all participants before their participation in the experiment.

### Participants

In total 58 young exercise trained females were recruited. All females belonged to university competitive athletic teams from both the track and field and badminton programs. To identify young females with and without a high sensitivity to cold, participants were required to complete a questionnaire (Table 1) developed by Nagashima et al. (2002). The questionnaire has ten questions directed at determining the cold sensitivity that individuals may typically experience under different conditions and or types of exposures. Participants were required to provide a yes or no response to each question. Respondents who answered yes to at least 7 of the 10 questions were defined as cold‐sensitive, whereas those who answered yes to two or less questions were defined as non‐cold‐sensitive. On this basis, 7 individuals were identified as cold‐sensitive while 7 were considered non‐cold‐sensitive (Control group). The remaining participants did not qualify for the study as they did not meet the specific inclusion criteria. This questionnaire has been shown to be a valid approach to differentiate between cold‐sensitive and non‐cold‐sensitive young females as defined by specific characteristics of cold sensation (Nagashima et al. 2002; Yamazaki and Sone 2017). None of the participants reported a history of chronic diseases, were taking prescription medication, or were smokers. Menstrual cycle was not controlled. The subjects' characteristics are presented in Table 2 and they did not differ between the groups. All subjects were instructed to abstain from taking over‐the‐counter medications for at least 48 h prior to any testing procedure, as well as refrain from consuming alcohol and caffeine and performing heavy exercise at least 12 h prior. To ensure that our participants were not heat acclimatized, all tests were performed in the autumn (October‐November).

**Table 1:** Interview questionnaire developed by Nagashima et al. (2002)

Do you or are you?
Sensitive to a reduction in environmental temperature? Feel colder in a cold environment than others do? Sometimes feel cold even in summer? Dislike being barefoot even in summer due to coldness? Feel cold in an air‐conditioned room in summer when most people feel comfortable? Need thicker clothes than others do? Need an electric blanket for better sleep in winter? Wear socks while sleeping in winter? Often wake up due to coldness or cold extremities in winter? Often have pain or color changes in the fingertips or toes due to bad circulation in cold?

**Table 2:** Participant characteristics

	Control	Cold‐sensitive
Number of participants	7	7
Age (years)	21 ± 2	22 ± 1
Height (m)	1.59 ± 0.05	1.62 ± 0.04
Body mass (kg)	52.3 ± 4.9	51.8 ± 6.3
Body surface area (m^2^)	1.53 ± 0.09	1.54 ± 0.10
Body mass index (kg m^−2^)	20.6 ± 1.1	19.7 ± 1.8
Peak oxygen uptake (ml kg^−1^ min^−1^)	47.7 ± 3.9	50.9 ± 5.1

All values are expressed as means ± standard deviation. There were no between‐group differences in any variables presented (*P* > 0.05).

### Peak oxygen uptake test

On a separate day and prior to the mild cold exposure test, participants performed an incremental cycling test using a self‐customized semi‐recumbent cycle ergometer (Model 874E, Monark, Stockholm, Sweden) to assess peak oxygen uptake. Initial work load was set at 60 W and was increased by 12 W every 1 min while the participant maintained a constant pedaling rate of 60 rpm. The test was terminated when the participant could no longer maintain a pedaling rate of >50 rpm or stopped due to volitional fatigue. During the test, the subjects breathed from a mask covering the mouth and nose. A mass‐flow sensor (hot‐wire type) and a gas‐sampling tube (the sampling volume rate was below 0.2 L min^−1^) were connected to the mask, and the expired volume and gases were analyzed using an electric gas flow meter (Model RM300i, Minato Medical Science, Japan). Peak oxygen uptake was taken as the highest rate of oxygen consumption measured over the final 1 min of the incremental exercise protocol. The test was performed in an environmental chamber (Fuji Medical Science Co., Ltd, Chiba, Japan) regulated to 25°C and 50% relative humidity.

### Mild cold exposure test

After a minimum of 2 days following the completion of the peak oxygen uptake test, the participants completed a mild cold exposure test, which was modified from the version developed by Nagashima et al. (2002). We determined that more than 48 h is necessary to minimize any carry‐over effect from the peak oxygen uptake test (e.g., muscle soreness, fatigue). The day prior to the test, all participants finished dinner by 8:00 pm, thereafter they were required to consume 500 mL water. On the experimental day, participants were required to arrive at the laboratory by 8:00 am and were required to abstain from consuming any food. On arrival the participants changed into shorts and sleeveless t‐shirts. The subjects did not wear shoes throughout the experiment including during exercise. Participants then entered an environmental chamber regulated at 29.5°C and a relative humidity of 50% (Fuji Medical Science Co., Ltd) where they rested in a semi‐recumbent position for 60 min during which time all equipment was affixed to the participant. Thereafter, baseline measurements were performed for an additional 40 min. Ambient temperature was then gradually reduced to 23.5°C [i.e., an ambient temperature considered to be within the range of normal indoor room temperatures (Kenny and Jay 2013)] over a 30 min period, and was maintained thereafter for the duration of the experiment. Once an ambient temperature of 23.5°C was reached, a 60‐min resting period was commenced. The participants then performed 30 min of moderate intensity semi‐recumbent cycling (equivalent to 50% of their predetermined peak oxygen uptake). We elected to use a moderate intensity of exercise equal to 50% of the participants predetermined peak oxygen uptake to ensure that all participants could complete the prolonged exercise bout without undue fatigue while eliciting a significant increase in core temperature. During the exercise bout, the participant's arms remained still while supported on tables located right next to both sides of participant. The exercise was followed by a 60 min recovery period with the participant remaining seated on the semi‐recumbent bike.

### Measurements

Subjective cold sensation for both the whole body and the extremities (i.e., fingers and toes) was obtained according to the method used by Nagashima et al. (2002). Specifically, the participants marked on a 15‐cm line rating scale for their level of cold sensation, with “cold” labeled 2.5 cm from the left end, whereas “not at all” was labelled 2.5 cm from the right end. Participants were allowed to mark beyond both ends of the 15‐cm rating scale if necessary (up to plus or minus 30 cm). This subjective cold sensation was obtained every 10 min throughout the experiment with the exception that measurements were performed every 5 min during exercise. Upon completion of the experimental session, one experimenter measured the length (cm) between the mark and the scale “not at all,” which was considered as the level of subjective cold sensation. Before the experiment, participants were instructed to differentiate whole‐body and extremity cold sensation. Previous studies using this scale have been successful in demonstrating greater cold sensation at rest in cold‐sensitive young females as compared to their non‐cold‐sensitive counterparts (Nagashima et al. 2002; Yamazaki and Sone 2017).

Core temperature was estimated using an esophageal temperature probe inserted via the nostril and positioned in the esophagus at a depth equivalent to one quarter of the subject's height. Skin temperature measured at eight sites (forehead, upper arm, hand, upper back, chest, thigh, calf, and toe) was used to assess mean whole‐body skin temperature based on the method of Hardy and Dubois (1938). Mean body temperature was calculated as 0.66 ×  esophageal temperature + 0.33 ×  mean skin temperature (Gagge and Nishi 1977). Mean extremity skin temperature was also evaluated as the mean of finger and toe skin temperatures. Esophageal and skin temperatures were obtained every 1 sec using self‐made copper constantan thermocouples. The data was stored in a computer through data logger system (WE7000, Yokogawa, Tokyo, Japan).

Forearm and finger skin blood flows were estimated from red blood cell flux using a laser‐Doppler flowmeter (ALF21, Advance, Tokyo, Japan). It was expressed as cutaneous vascular conductance, which was calculated as the laser‐Doppler perfusion units divided by mean arterial pressure. Values were normalized to the baseline values as assessed using the last 20 min of the 40‐min baseline resting period at 29.5°C. Arterial blood pressures were measured every 1 min via an automated sphygmomanometer (STBP‐780; Nippon Colin, Tokyo, Japan). Heart rate was recorded every 5 sec via a heart rate monitor (HR monitor Vantage NV, POLAR, Kempele, Finland). An automated metabolic system (Model RM300i, Minato Medical Science) was employed to measure minute ventilation, oxygen uptake, carbon dioxide output, and respiratory exchange ratio at 30‐sec sampling rate. Metabolic rate was subsequently estimated from oxygen uptake and respiratory exchange ratio (Weir 1949), and was normalized to body surface area (kcal m^−2^). During the exercise, perceived exertion using a 6‐ to 20‐point rating scale (Borg 1975) was recorded every 5 min.

### Data analyses

All data used for time‐dependent data analysis were averaged at each 10 min interval. In addition, whole‐body cold sensation at a mean whole‐body skin temperature of 31.5, 32.5, and 33.5°C was evaluated. Similarly, cold sensation at the extremities at a mean extremity skin temperature of 26, 28, 30, and 32°C was also assessed. For the data analysis evaluating cold sensation at these predefined skin temperatures, measurements of cold sensitivity were taken once temperatures remained stable for 10 min (5 min during exercise) and did not vary more than ±0.3°C. The change in esophageal temperature was assessed from the last 10‐min of preexercise resting at 23.5°C (immediately prior to the onset of exercise) to the end of exercise. During exercise, the relationship between cutaneous vascular conductance and the change in esophageal temperature was evaluated using the average value recorded over a 30‐sec period. From this relationship, two regression lines were derived with a least square fitting approach. The change in esophageal temperature wherein the two regression lines crossed was employed to identify the core temperature threshold at which an increase in cutaneous vasodilation occurred. The slope of the second regression line was used to measure the thermosensitivity of cutaneous vasodilation. The threshold and thermosensitivity of cutaneous vasodilation was also evaluated using mean body temperature. The regression lines and threshold were assessed with the aid of a computer algorithm developed by our laboratory.

### Statistical analyses

**Figure 1:**
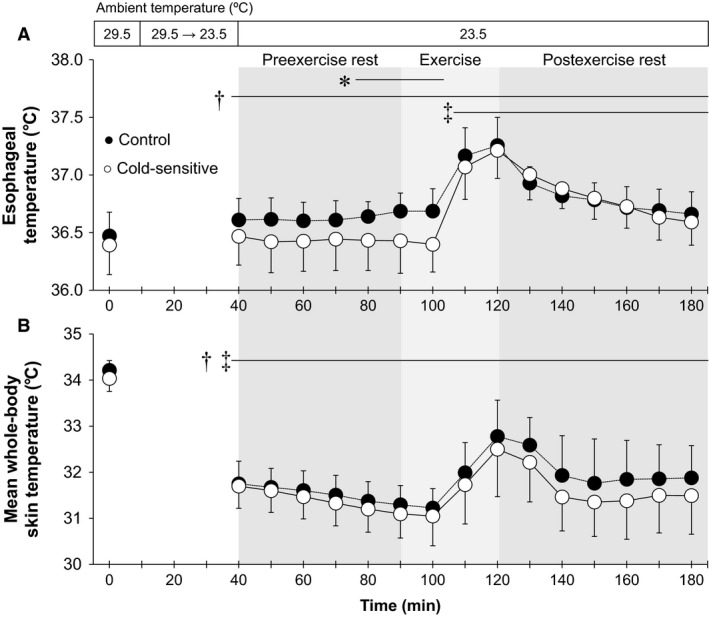
Time‐course changes in esophageal (A) and mean whole‐body skin (B) temperatures. *, between groups (*P* ≤ 0.05); †, versus 29.5°C baseline in the Control group (*P* ≤ 0.05); ‡, versus 29.5°C baseline in the Cold‐sensitive group (*P* ≤ 0.05). Data are mean ± standard deviation (*n* = 7 for each group).

**Figure 2:**
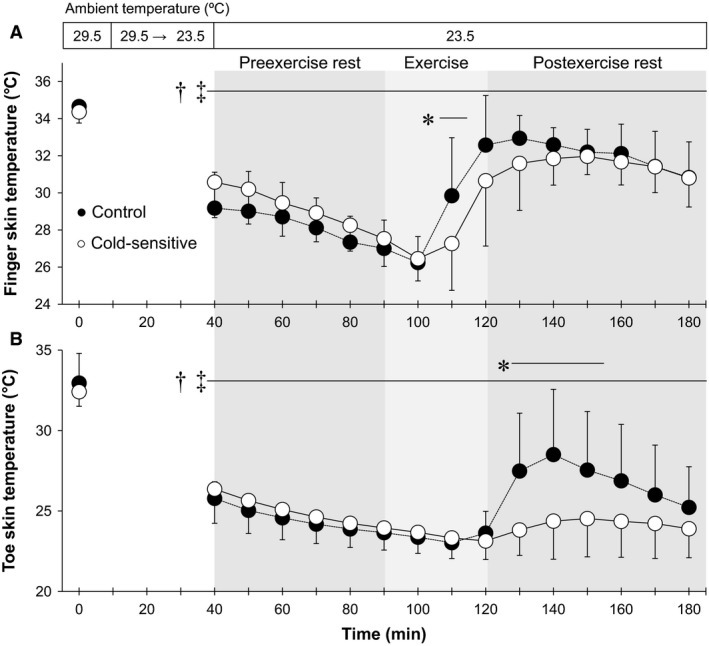
Time‐course changes in finger (A) and toe (B) skin temperatures. *, between groups (*P* ≤ 0.05); †, versus 29.5°C baseline in the Control group (*P* ≤ 0.05); ‡, versus 29.5°C baseline in the Cold‐sensitive group (*P* ≤ 0.05). Data are mean ± standard deviation (*n* = 7 for each group).

**Figure 3:**
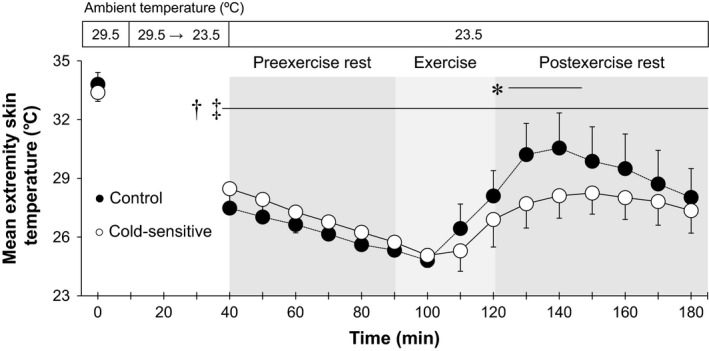
Time‐course changes in mean extremity skin temperature (an average of finger and toe skin temperatures). *, between groups (*P* ≤ 0.05). †, versus 29.5°C baseline in the Control group (*P* ≤ 0.05); ‡, versus 29.5°C baseline in the Cold‐sensitive group (*P* ≤ 0.05). Data are mean ± standard deviation (*n* = 7 for each group).

**Figure 4:**
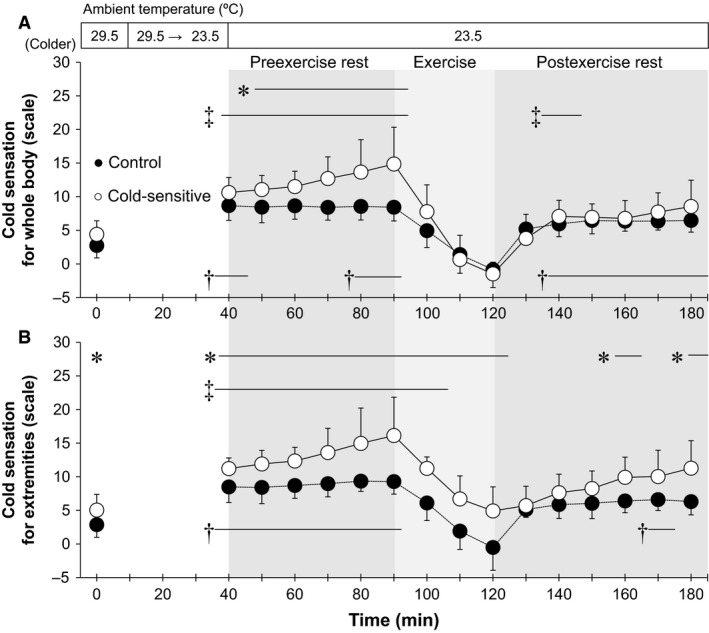
Time‐course changes in cold sensation for whole‐body (A) and extremities (B). *, between groups (*P* ≤ 0.05). †, versus 29.5°C baseline in the Control group (*P* ≤ 0.05); ‡, versus 29.5°C baseline in the Cold‐sensitive group (*P* ≤ 0.05). Data are mean ± standard deviation (*n* = 7 for each group).

**Figure 5:**
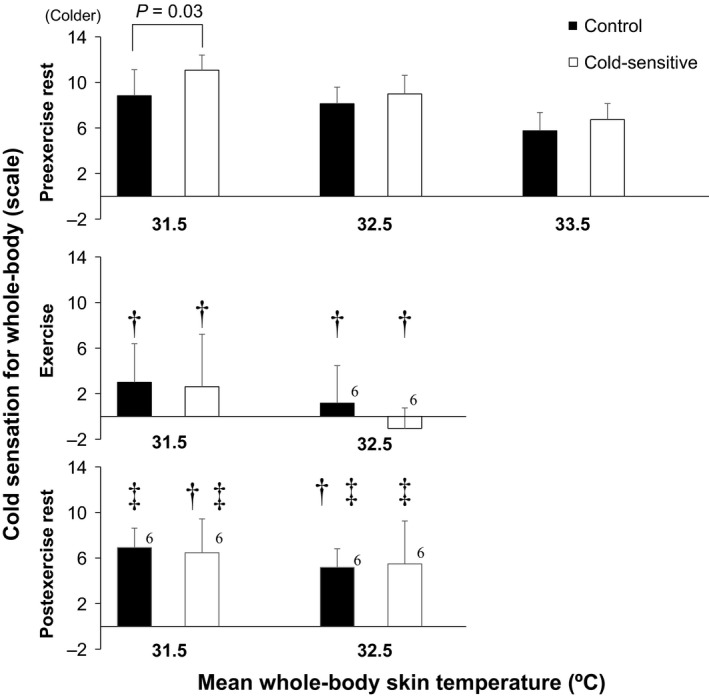
Whole‐body cold sensation at given mean whole‐body skin temperatures. †, versus preexercise rest (*P* ≤ 0.05); ‡, versus exercise (*P* ≤ 0.05). Data are mean ± standard deviation (*n* = 7 for each group). Number adjacent to bar indicates the number of remaining subjects.

**Figure 6:**
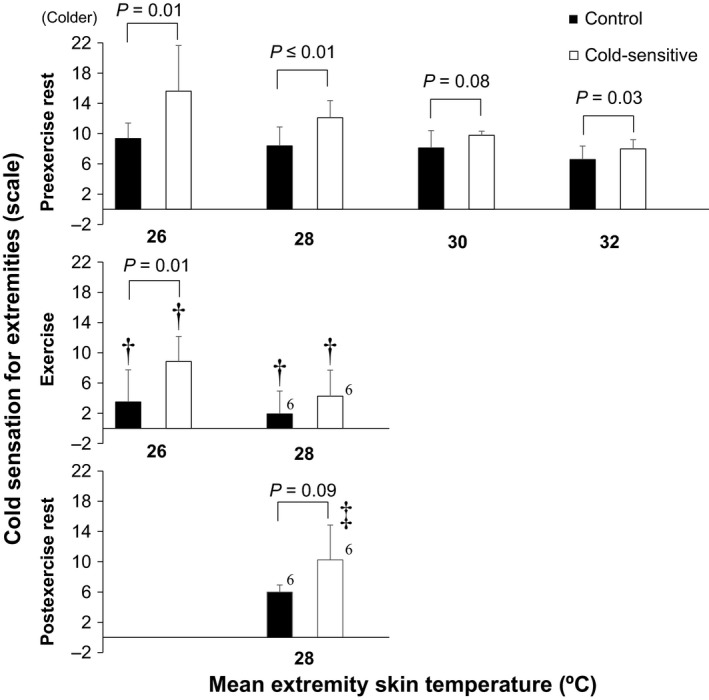
Extremity cold sensation at the different mean extremity skin temperatures. †, versus preexercise rest (*P* ≤ 0.05). ‡, versus exercise (*P* ≤ 0.05). Data are mean ± standard deviation (*n* = 7 for each group). Number adjacent to bar indicates the number of remaining subjects.

Data are presented as mean ± standard deviation. Parametric analyses were performed on all variables with the exception that cold sensation was analyzed using nonparametric tests. Time‐dependent data (Figs. 1, 2, 3, 4, Table 3) were analyzed using a two‐way mixed‐design analysis of variance with two factors of group (Control and Cold‐sensitive) and time (29.5°C baseline and every 10 min during exposure to room temperature conditions of 23.5°C). Cold sensation for the whole‐body evaluated at a mean whole‐body skin temperature of 31.5°C (Fig. 5) was analyzed by a two‐way mixed‐design analysis of variance with two factors of group (Control and Cold‐sensitive) and stage (Preexercise rest, Exercise, Postexercise rest). The same analysis was performed at a mean whole‐body skin temperature of 32.5°C (Fig. 5). Cold sensation for the extremities evaluated at a mean extremity skin temperature of 28°C (Fig. 6) was analyzed by a two‐way mixed‐design analysis of variance with two factors of group (Control and Cold‐sensitive) and stage (Preexercise rest, Exercise, Postexercise rest). The same analysis was conducted at a mean extremity skin temperature of 26°C (data analysis was performed for preexercise rest and exercise only, as the most of subjects showed a mean skin temperature of >26°C during the postexercise period). When a main effect or an interaction was detected, *posthoc* comparisons were performed using *t*‐tests, with *P* values adjusted by the modified Bonferroni procedures [i.e., Holm's (Gordon and Salzman 2008) or Hochberg's (Hochberg 1988) procedure]. In addition, *t*‐tests were used to compare variables where applicable (e.g., Table 2). A value of *P* ≤ 0.05 was considered statistically significant. Statistical analyses were performed using the software package SPSS 24 (IBM, Armonk, NY).

**Table 3:** Time‐course changes in cardiovascular and metabolic variables

	29.5°C	23.5°C						
	Baseline	Rest	Ex10	Ex20	Ex30	Rec20	Rec40	Rec60
Heart rate, beats min^−1^								
Control	61 ± 8	61 ± 7	115 ± 11^a^	126 ± 11^a^	129 ± 10^a^	72 ± 7^a^	69 ± 8^a^	67 ± 9^a^
Cold‐sensitive	58 ± 10	58 ± 11	116 ± 17^b^	125 ± 20^b^	127 ± 19^b^	68 ± 18	66 ± 15	64 ± 13
Mean arterial pressure, mmHg								
Control	78 ± 8	83 ± 6	96 ± 10^a^	95 ± 8^a^	94 ± 8^a^	82 ± 7^a^	82 ± 5^a^	81 ± 10
Cold‐sensitive	81 ± 4	85 ± 4^b^	91 ± 6^b^	94 ± 5^b^	93 ± 4^b^	84 ± 3	81 ± 3	81 ± 4
Forearm cutaneous vascular conductance, %baseline								
Control	100	50 ± 14^a^	81 ± 36	174 ± 133	252 ± 181	97 ± 57	77 ± 37	77 ± 46
Cold‐sensitive	100	49 ± 18^b^	77 ± 26	180 ± 50^b^	321 ± 89^b^	72 ± 9^b^	79 ± 32	85 ± 39
Forearm cutaneous perfusion, perfusion units								
Control	0.06 ± 0.02	0.03 ± 0.01^a^	0.06 ± 0.02	0.11 ± 0.08	0.16 ± 0.10	0.06 ± 0.03	0.05 ± 0.02	0.05 ± 0.03
Cold‐sensitive	0.05 ± 0.02	0.03 ± 0.01^b^	0.04 ± 0.01	0.11 ± 0.04^b^	0.20 ± 0.09^b^	0.04 ± 0.01^b^	0.04 ± 0.01	0.04 ± 0.02
Finger cutaneous vascular conductance, %baseline								
Control	100	22 ± 7^a^	28 ± 13^a^	76 ± 37	91 ± 37	79 ± 19	68 ± 16^a^	54 ± 23^a^
Cold‐sensitive	100	25 ± 10^b^	21 ± 7^b^	58 ± 30^b^	78 ± 41^b^	65 ± 7^b^	67 ± 13^b^	55 ± 17^b^
Finger cutaneous perfusion, perfusion units								
Control	0.20 ± 0.07	0.05 ± 0.02^a^	0.06 ± 0.02^a^	0.17 ± 0.09	0.20 ± 0.08	0.16 ± 0.05	0.13 ± 0.03^a^	0.11 ± 0.06^a^
Cold‐sensitive	0.20 ± 0.10	0.05 ± 0.02^b^	0.05 ± 0.01^b^	0.14 ± 0.06^b^	0.20 ± 0.09	0.14 ± 0.08^b^	0.14 ± 0.09^b^	0.11 ± 0.09^b^
Metabolic rate, kcal m^−2^								
Control	32 ± 2	31 ± 5	186 ± 52^a^	196 ± 41^a^	193 ± 32^a^	34 ± 3	31 ± 2	31 ± 3
Cold‐sensitive	31 ± 2	33 ± 3	200 ± 25^b^	212 ± 30^b^	208 ± 22^b^	32 ± 2	30 ± 2	29 ± 2

Data are mean ± standard deviation (*n* = 7 for each group). Rest, preexercise rest, Ex, exercise; Rec, postexercise recovery; numbers adjacent to Ex and Rec indicate minutes into each period.

a Versus 29.5°C baseline in the Control group (*P* ≤ 0.05);

b Versus 29.5°C baseline in the Cold‐sensitive group (*P* ≤ 0.05). There were no between‐group differences throughout for all variables (all *P* > 0.05).

## Results

### Body temperatures

During exposure to an ambient temperature of 23.5°C in comparison to baseline at 29.5°C, esophageal temperature increased during preexercise rest in the Control group only, whereas it increased during and following an exercise‐induced heat stress in both groups (all *P* ≤ 0.05, Fig. 1A). All skin temperature variables decreased during exposure to an ambient temperature of 23.5°C relative to 29.5°C throughout the experiment (Figs. 1B, 2, 3). Cold‐sensitive group showed a lower esophageal temperature relative to the Control group over the last 20 min of the preexercise rest period (e.g., at the last 10 min, 36.4 ± 0.3 vs. 36.7 ± 0.2°C, *P* ≤ 0.05) as well as the first 10 min of exercise (Fig. 1A). The change in esophageal temperature from the last 10‐min of preexercise rest to the end of exercise at 23.5°C tended to be greater in the Cold‐sensitive versus Control groups (0.78 ± 0.25 vs. 0.57 ± 0.27°C, *P* = 0.08). Further, the Cold‐sensitive group showed a lower finger skin temperature at 20‐min into exercise (27.3 ± 2.5 vs. 29.8 ± 3.1°C, *P* ≤ 0.05, Fig. 2A), and a lower toe skin temperature (difference of ~3–4°C, Fig. 2B) and mean extremity skin temperature (difference of ~2.5°C difference, Fig. 3) at 10‐, 20‐ or 30‐min into postexercise recovery (all *P* ≤ 0.05). Beyond these differences, body temperatures did not differ between groups (all *P* > 0.05).

### Cold sensation

Cold sensation for the whole‐body and the extremities increased during exposure to an ambient temperature of 23.5°C during both pre or postexercise rest in both groups as compared to resting at 29.5°C (all *P* ≤ 0.05, Fig. 4). However, cold sensations at 20‐and 30‐min of exercise were similar to those observed during resting with an ambient temperature of 29.5°C for both groups (all *P* > 0.05, Fig. 4). During exposure to temperate ambient conditions (i.e., 23.5°C), cold sensation was greater in the Cold‐sensitive versus Control groups during preexercise rest for the whole‐body, whereas responses for the extremities were greater throughout the experiment (all *P* ≤ 0.05) except between 10‐ 20‐, 30‐, and 50‐min following cessation of exercise (both *P* > 0.05) (Fig. 4). Similar between‐group differences were measured for cold sensations of the whole‐body and extremities as determined for a given mean whole‐body and extremity skin temperatures (Figs. 5, 6). Cold sensation assessed for the whole‐body and the extremities increased as skin temperature decreased (Figs. 5, 6). At given skin temperatures, it was reduced during exercise and remained lower throughout the postexercise period; however, for the postexercise period, this was limited to whole‐body cold sensation only (Figs. 5, 6, *P* ≤ 0.05).

### Secondary variables

There were no group differences in heart rate, mean arterial pressure, forearm and finger cutaneous vascular conductance and perfusion, metabolic rate (Table 3), as well as ratings of perceived exertion measured during exercise (all *P* > 0.05). Metabolic rate during preexercise rest remained unchanged from values at 29.5°C to the end of ambient exposure to 23.5°C in both groups (data not shown), inferring that shivering did not occur under these conditions. The change in esophageal temperature threshold at which an increase in cutaneous vascular conductance occurred did not differ between the Control and Cold‐sensitive groups (both *P* > 0.05, Table 4). Also, the thermosensitivity of the cutaneous vascular conductance response, as defined by the slope relating cutaneous vascular conductance with the change in esophageal temperature, was similar between the Control and Cold‐sensitive groups (both *P* > 0.05, Table 4). No between‐group differences in the threshold and thermosensitivity were also observed using mean body temperature (Table 4).

**Table 4:** Forearm and finger cutaneous vascular conductance during exercise in response to the change in esophageal and mean body temperatures

	Forearm cutaneous vascular conductance		Finger cutaneous vascular conductance	
	∆Threshold, °C	Slope, %baseline °C	∆Threshold, °C	Slope, %baseline °C
Esophageal temperature				
Control	0.33 ± 0.28	513 ± 351	0.20 ± 0.35	128 ± 104
Cold‐sensitive	0.43 ± 0.32	850 ± 330	0.23 ± 0.31	89 ± 49
Mean body temperature				
Control	0.43 ± 0.29	374 ± 324	0.25 ± 0.36	75 ± 32
Cold‐sensitive	0.48 ± 0.51	564 ± 277	0.23 ± 0.36	80 ± 63

Data are mean ± standard deviation (*n* = 7 for each group). ∆Threshold, the change in esophageal and mean body temperatures from the last 10‐min of preexercise rest at 23.5°C to the threshold temperature for increase in cutaneous vascular conductance. Slope, the slope relating cutaneous vascular conductance with the change in esophageal and mean body temperatures from the last 10‐min of preexercise rest at 23.5°C. No between‐group differences were observed for all of the data presented (all *P* > 0.05).

## Discussion

We showed that during exposure to a temperate ambient condition of 23.5°C, young exercise trained females with a high sensitivity to cold demonstrated an augmented whole‐body cold sensation during preexercise rest compared to their non‐cold‐sensitive counterparts. However, this response was diminished during and following an exercise‐induced heat stress. We also observed a greater cold sensation on the extremities in the cold‐sensitive exercise trained females at rest, and this response persisted during and following exercise.

### Rest

Cold sensation for both the whole‐body and the extremities increased in both groups under resting conditions in response to a decrease in the level of heat stress as assessed by decreasing ambient temperature from 29.5 to 23.5°C (Fig. 4). This was paralleled by a greater reduction in skin temperatures in both groups (Figs. 1B, 2, 3) which occurred without a concomitant decrease in core temperature (Fig. 1A). Taken together, our findings demonstrate that the increased cold sensation appears to occur secondary to reductions in skin temperatures. These findings are consistent with previous studies that showed that skin temperature is a main factor determining thermal sensation during cold exposure under resting conditions (Gagge et al. 1969; Mower 1976; Katsuura et al. 1998). Furthermore, Nagashima et al. (2002) demonstrated that cold sensation on the whole‐body and the extremities were highly correlated with mean whole‐body skin temperature and extremity skin temperature (i.e., finger skin temperature), respectively during exposure to temperate ambient conditions at rest.

During preexercise rest, we showed that cold sensation of both the whole‐body and the extremities under an ambient temperature of 23.5°C was greater in the Cold‐sensitive relative to the Control group (Fig. 4); a response which is consistent with previous reports (Nagashima et al. 2002; Yamazaki 2015). This augmentation was observed without differences in skin temperatures between groups (Figs. 1B, 2, 3). In context of our study findings, our results suggest that young exercise trained females who are sensitive to cold exhibit greater cold sensation for a given peripheral thermal input as defined by skin temperature. Previous studies (Nagashima et al. 2002; Yamazaki 2015) reported similar findings wherein they showed a greater cold sensitivity in response to decreasing skin temperatures in untrained young females. Given cold‐sensitive young females have been observed in both untrained (Nagashima et al. 2002; Yamazaki 2015) and trained (the current study) individuals, training status may have a minimal influence on the augmented cold sensation measured in cold‐sensitive young females. To more clearly delineate if fitness level modulates cold sensation, future studies directly assessing cold sensation in both trained and untrained females or in response to an exercise training program are warranted.

### Exercise

A significant increase in core and mean skin temperatures, and therefore body warming occurred during exercise (Fig. 1). We showed that cold sensation of the whole‐body and extremities was attenuated during exercise in both groups (Fig. 4). Studies suggested that thermal sensation is mainly regulated by skin temperatures even during an exercise‐induced heat stress (Gagge et al. 1969; Schlader et al. 2011). Hence, the exercise‐induced reduction in cold sensation may be due to increased skin temperatures (Figs. 1B, 2, 3). However, the reduction in cold sensation during exercise was also evident at the same skin temperatures (Figs. 5, 6) which may indicate that factor(s) other than skin temperature may also be involved. A recent study by Gerrett et al. (2015) showed that exercise *per se* reduces cold sensation. They postulated that this could be due to an analgesic effect associated with exercise which causes a decrease in the transmission of sensory information. Further study is warranted to evaluate the mechanisms underpinning the exercise‐induced reductions in cold sensation.

In keeping with our study hypothesis, we observed no differences in whole‐body cold sensation during exercise between groups (Figs. 4A, 5). Our findings indicate that exercise attenuates the elevated whole‐body cold sensation in young exercise trained females who are cold‐sensitive. However, in contrast to our hypothesis, cold sensation of the extremities during exercise remained elevated in the Cold‐sensitive relative to the Control group (Fig. 4B), even sensation was assessed at the same mean extremity skin temperatures (Fig. 6). It may be that the blunting effect of exercise on cold sensation differs depending on the skin site (i.e., a regional effect) such that its effect on the extremities is relatively smaller than that for the whole‐body. Alternatively, this difference may be due to differences in lower extremity versus mean whole‐body skin temperatures during exercise (26–28 vs. 31.5–32.5°C) (Figs. 1B, 3). Although speculative, the greater reduction in skin temperature may override the analgesic effect associated with exercise. Future studies are required to assess this possibility.

We showed that toe skin temperature remained unchanged during exercise (Fig. 2B) despite the fact that finger skin temperature increased throughout exercise (Fig. 2A). An increase in finger skin temperature could reflect an increase in heat loss associated with an elevation in skin perfusion (as assessed by finger cutaneous vascular conductance) (Table 3) secondary to an increase in core and skin temperatures. Differences between toe and finger temperatures were also reported in a previous work by Brajkovic et al. (1998) wherein they showed that heating the torso increased finger but not toe skin temperature. In contrast to toe temperature, we showed that calf skin temperature increased by >1.0°C in both groups. Hence, the attenuation in cutaneous perfusion, if any, did not appear to occur over the lower limbs. This may reflect different sympathetic mechanisms underpinning the regulation of cutaneous blood flow between glabrous (e.g., toe) and nonglabrous (e.g., calf) skin (Johnson et al. 2014). Alternatively, since the participants in our study were pedaling during exercise, pressure applied to the foot may have restricted toe skin perfusion only thereby contributing to the unchanged toe skin temperature.

### Postexercise rest

To the best of our knowledge, there are no previous studies that have examined thermal sensation during the postexercise recovery period. While we showed that core and mean skin temperatures declined at the cessation of exercise, they remained elevated during the early stages of recovery (<30 min); an indication that a significant residual heat load remained within the body (Kenny and McGinn 2017). We showed that the exercise‐induced attenuation of whole‐body cold sensation at a given mean whole‐body skin temperature persisted into the recovery period for both groups (Fig. 5). The underlying mechanism(s) for this response is unclear. Given the participants remained inactive during this period, any residual analgesic effect associated with exercise would likely have subsided in the early stages of recovery. Clearly, more work is required to elucidate the mechanisms underlying the blunted whole‐body cold sensation during the postexercise recovery period.

In contrast to whole‐body cold sensation, the exercise‐induced reduction in cold session of the extremities was diminished postexercise, returning to the preexercise levels (Fig. 6). This suggests that the pattern of response for cold sensation differs between the whole‐body and the extremities during the postexercise period. This is supported by our observation that during postexercise recovery, there were no‐between group differences in whole‐body cold sensation (Fig. 4A), whereas cold sensation of the extremities was greater in the Cold‐sensitive compared to the Control group (Fig. 4B). Thus, young trained females who are sensitive to cold exhibit augmented cold sensation of the extremities but not of the whole‐body following an exercise‐induced heat stress.

### Individual differences in cold sensation

While we observed large differences in cold sensation between groups, our study was not designed to assess the mechanism(s) underpinning this response. Our results, however, show that the cardiovascular (including the heat loss response of cutaneous vasodilation during exercise) and metabolic responses did not differ between the groups (Table 3). Also, age, body size, and aerobic capacity were all matched between groups (Table 2). The greater cold sensation in the Cold‐sensitive group could be due to a greater excitation of temperature‐sensitive neurons in the brain for a given peripheral input and/or greater temperature‐sensitive receptor excitation in the skin at a given cold stimuli. Regarding the latter, Yamazaki and Sone (2017) showed that the transient receptor potential melastatin‐8 is not likely involved in the augmented cold sensation as assessed using menthol (a transient receptor potential melastatin‐8 agonist) applied to the skin. Additional research is required to elucidate the mechanism(s) underpinning the individual variations in cold sensation.

### Influence of core temperature on cold sensation

Although cold sensation appears to be mainly mediated by reductions in skin temperatures (Chatonnet and Cabanac 1965; Gagge et al. 1969; Mower 1976; Attia 1984; Katsuura et al. 1998; Yao et al. 2007; Zhang et al. 2010; Schlader et al. 2011), we do not know if, and to what extent, core temperature affected cold sensation in the current study. We showed that esophageal temperature in the Cold‐sensitive group was lower as compared to the Control group during exposure to an ambient temperature of 23.5°C (Fig. 1A). This may be related to the greater whole‐body cold sensation in the Cold‐sensitive females under these conditions (Fig. 4A). This difference in esophageal temperature was not a result of differences in metabolic rate (and therefore heat production) associated with an increase in shivering. This is supported by our observation that the metabolic rate during preexercise rest at 23.5°C remained unchanged in both groups. Further, although absolute esophageal temperature was similar between the groups at the end of exercise (Fig. 1A), we showed that the Cold‐sensitive group tended to demonstrate a greater change in esophageal temperature relative to their non‐cold‐sensitive counterparts (i.e., 0.78 ± 0.25 vs. 0.57 ± 0.27°C, *P* = 0.08) (i.e., evaluated as the change in esophageal temperature from the last 10‐min of preexercise rest at 23.5°C). Since we did not observe differences in either metabolic rate (Table 3) or heat loss associated with cutaneous vasodilation (Tables 3, 4) between the groups, the greater increase in the change in esophageal temperature measured in the Cold‐sensitive group may be attributed to a reduction in heat loss associated with an attenuation of the sweating response. This response requires further examination.

### Limitations

There are five limitations to note in this study. Firstly, we did not explicitly define the extremes of our scale (i.e., +30 cm and −30 cm from “not at all”). Hence, it is not possible to determine the extent or degree to which participants felt cold if they marked at or near +30 cm. Secondly, we tested young Japanese females only. Given that ethnicity may be a factor affecting thermal sensation (Wang et al. 2010), our results may not reflect responses in other populations such as Caucasians. Thirdly, we did not control for menstrual cycle phase. Although a recent study showed that thermal sensation and pleasantness during exposure to room temperature conditions of 23.5°C did not differ across menstrual cycle phase (Matsuda‐Nakamura et al. 2015), another study reported that sex hormones can influence cold perception (Cankar et al. 2016) as well as heat loss response of cutaneous vasodilation (Kuwahara et al. 2005). Thus, we cannot exclude the possibility that menstrual cycle phase may be a confounding factor in our study findings. Fourthly, we did not conduct a time‐control trial without the inclusion of an exercise bout. Hence, we do not know how the 150‐min prolonged exposure to 23.5°C influenced cold sensation and the associated physiological responses, and the extent to which these responses were altered during and following exercise. Lastly, we did not assess thermal comfort. Thermal perception has two components: thermal comfort and sensation. The factors regulating thermal sensation differs from that of thermal comfort such that thermal comfort is greatly influenced by core temperature (Cabanac et al. 1971; Scarperi and Bleichert 1983), whereas the majority of studies have shown that changes in core temperature have a minimal effect on thermal sensation (Chatonnet and Cabanac 1965; Gagge et al. 1969; Mower 1976; Attia 1984; Katsuura et al. 1998; Yao et al. 2007; Zhang et al. 2010; Schlader et al. 2011). It remains to be seen whether there are individual variations in thermal comfort in young trained females during and following an exercise‐induced increase in core temperature.

### Perspectives and significance

Our findings show that young exercise trained females who are sensitive to cold exhibit augmented cold sensation on the extremities only, and not the whole‐body, during and following an exercise‐induced heat stress. Hence, warming the fingers and toes (e.g., wearing socks and gloves) during and following exercise may be an effective strategy to minimize an augmented cold sensation in these individuals. Furthermore, warming the fingers and toes during and after a warm‐up for exercise may mediate changes in cold sensation, and therefore general comfort level, that might ultimately contribute to better exercise performance especially when exposed to a cold environment.

## Conclusions

We show that during exposure to a non‐heat stress ambient temperature of 23.5°C, young trained females who are sensitive to cold have a greater whole‐body cold sensation during rest. However, this response is diminished during and following an exercise‐induced heat stress. In contrast, young trained cold‐sensitive females exhibit augmented cold sensation of the extremities during rest, as well as during and following exercise.

## Conflict of Interest

None.
